# Repeat epidural blood patch at the level of unintentional dural puncture and its neurologic complications: a case report

**DOI:** 10.1186/s40981-019-0232-3

**Published:** 2019-02-28

**Authors:** Kentaro Iga, Takeshi Murakoshi, Airi Kato, Keiichiro Kato, Shuhei Terada, Hiroko Konno, Shingo Irikoma, Takashi Suzuki, Mitsuru Matsushita, Yoshie Toba

**Affiliations:** 1 Division of Perinatology, Fetal Diagnosis and Therapy, Maternal and Perinatal Care Center, Seirei Hamamatsu General Hospital, 2-12-12 Sumiyoshi, Hamamatsu City, Shizuoka Japan; 2 Department of Anesthesiology, Seirei Hamamatsu General Hospital, 2-12-12 Sumiyoshi, Hamamatsu City, Shizuoka Japan

**Keywords:** Arachnoiditis, Epidural blood patch, Post-dural-puncture headache, Subdural hematoma

## Abstract

**Background:**

Autologous epidural blood patch (AEBP) is effective for post-dural-puncture headache (PDPH). In some cases, repeat procedures are required for complete cure. In rare instances, severe adverse effects can occur. We present a case of neurologically complicated AEBPs, one of which was performed at the interspace of unintentional dural puncture (UDP).

**Case presentation:**

A 40-year-old primigravida sustained UDP at the L2–3 interspace during combined spinal-epidural anesthesia for a scheduled cesarean section. She developed PDPH and underwent a single AEBP at L3–4. The PDPH recurred and she required another AEBP at L2–3, after which she reported radicular pains. A diagnosis of subdural hematoma and adhesive arachnoiditis was made. Her symptoms partially resolved in the following months.

**Conclusion:**

It may be prudent to reconsider the use of repeated AEBP and to avoid the interspace of UDP. A thorough evaluation is warranted to exclude treatable lesions when adverse effects occur.

## Background

Unintentional dural puncture (UDP) by epidural needle complicates the care of 1.5% of obstetric patients; post-dural-puncture headache (PDPH) is a common complication [1]. Rapid treatment for PDPH is indicated in the obstetric population because severe symptoms can prevent mother-neonate interactions [2]. Treatment options include autologous epidural blood patch (AEBP), in which blood is injected into the epidural space; this may alleviate symptoms by causing continued tamponade [3], adhering to the dural sac for an extended period of time at extended vertebral levels [4, 5], thereby restoring intracranial pressure and reducing cerebral vasodilation. Multiple courses of AEBP may be necessary [6–9].

Rare, severe neurological complications of AEBP have been reported [9], in addition to common issues, including post-procedural pain in the low back, buttock, or leg [9]. Inadvertent injection of blood into the subarachnoid space may result in subdural hematoma and adhesive arachnoiditis, which may lead to permanent nerve damage [10]. Subdural blood causes adhesive arachnoiditis by releasing free radicals, which inflame pia and arachnoid mater, causing localized fibrosis [11]. In AEBP, blood may enter the subdural space by direct injection or through another dural hole caused by multiple attempts to locate the epidural space [12]. These conditions can be suspected clinically and identified by magnetic resonance imaging (MRI) [10]. Early diagnosis is important because these conditions may mimic others that require expedited interventions, including abscess formation [13].

We present a case of subacute subdural hematoma and adhesive arachnoiditis following two courses of AEBP, one of which was performed at the interspace of UDP. This condition was diagnosed clinically and identified using MRI.

## Case presentation

A 40-year-old Japanese primigravida with American Society of Anesthesiologists Performance Status 1 was scheduled for an elective cesarean section because of a low-lying placenta at 38 weeks of gestation. Her past, pertinent medical history was unremarkable. Combined spinal-epidural anesthesia (CSEA) was planned for the surgery. The patient was placed in a right lateral recumbent position. Eighty-three percent alcohol with 0.5% chlorhexidine was used for skin preparation. A 16-gauge CSEcure® needle (Smiths Medical Japan, Tokyo, Japan) was inserted at the L2–3 interspace. Loss of resistance to saline was noted at 3.3 cm using a median approach. A 27-gauge pencil point needle was introduced by 5 mm. On advancing the spinal needle, the patient experienced radiating pain in her right leg, which, unfortunately, caused her to move. At this time, we identified UDP with a constant stream of clear cerebrospinal fluid. The epidural needle was immediately removed. CSEA was again performed at the L3–4 interspace using an identical 16-gauge needle, with a loss of resistance to saline at 3.0 cm followed by an uneventful needle-through-needle spinal tap. We injected 8 mg of hyperbaric bupivacaine and 20 μg of fentanyl intrathecally, and placed a 17-gauge Perifix® catheter (B Braun, Tokyo, Japan) epidurally. There were no signs of CSF backflow nor blood backflow through the needle or the catheter. CSEA resulted in an inadequate block at the level of Th12. A decision was made to perform supplemental epidural anesthesia at the Th12-L1 interspace using a 17-gauge Uniever® needle (Unisys, Tokyo) with 6 mL of epidural 0.75% ropivacaine, which yielded adequate anesthesia for the operation, without complications. The remainder of the delivery was uneventful.

**Fig. 1:**
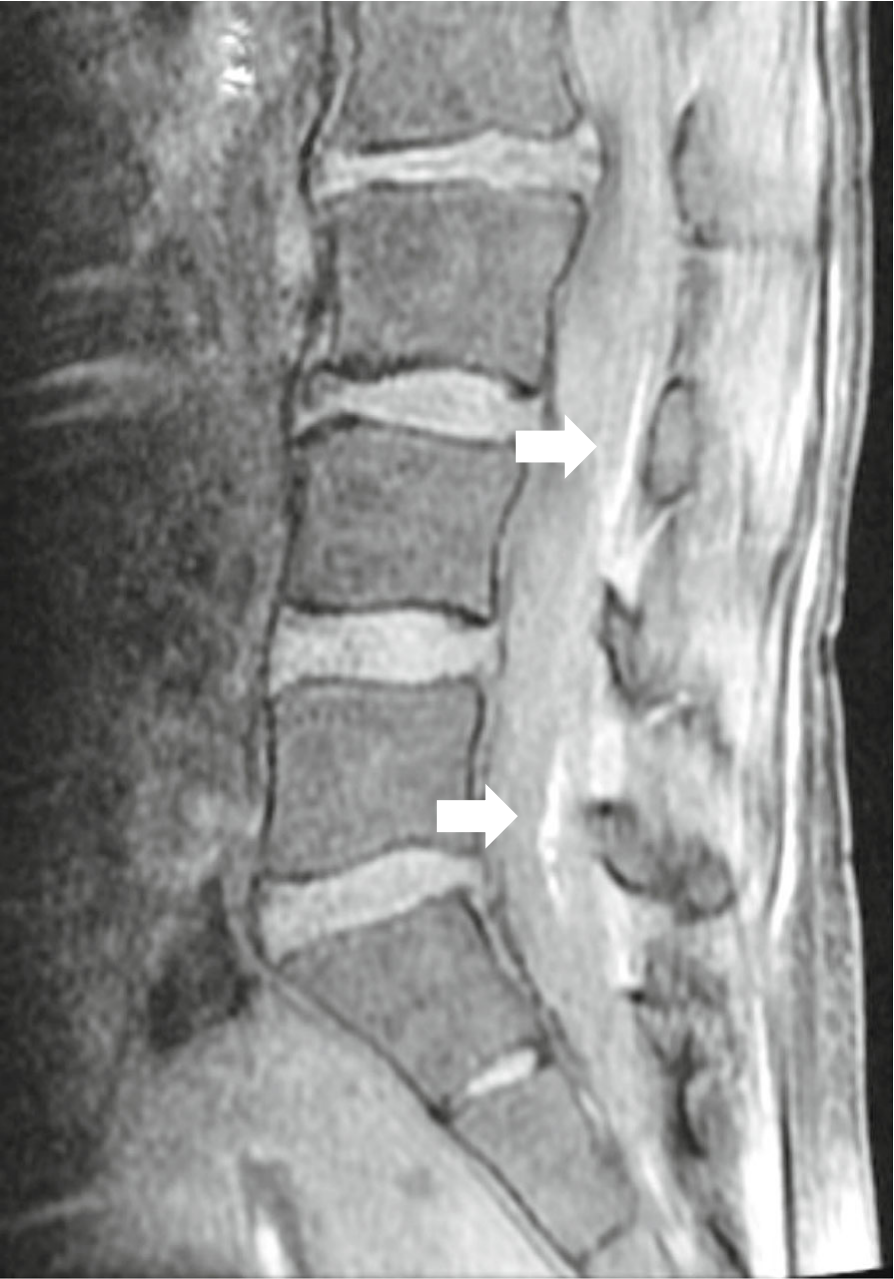
A parasagittal T1-weighted image. The magnetic resonance image shows subdural blood collection from the epidural blood patch. White arrows indicate intrathecal blood. These subdural clots were identified as two separate hematomas on MRI

**Fig. 2:**
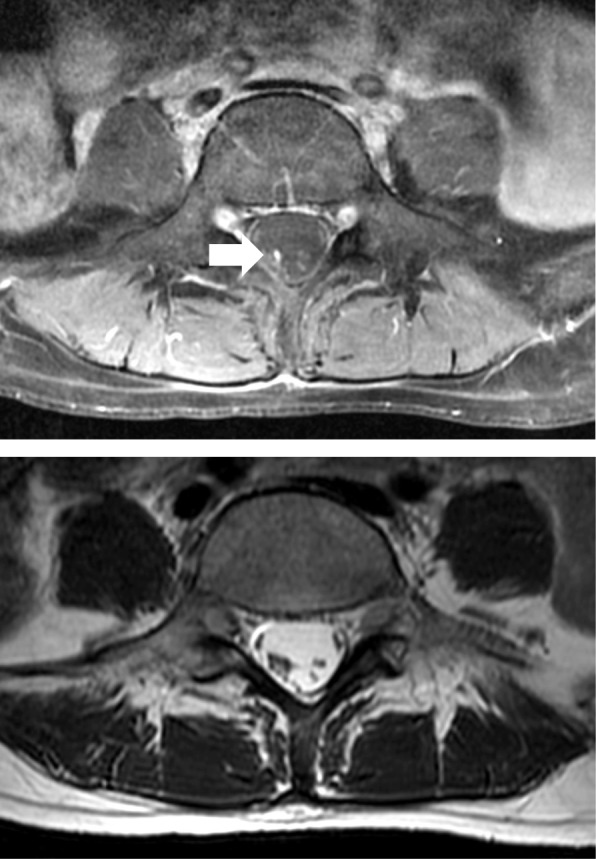
An axial gadolinium-enhanced T1-weighted image at the level of L5 and a T2-weighted image at the same level. These images show clumpy cauda equina fibers within dura mater. White arrow indicates a cauda equina aggregated posteriorly, with identifiable blood in a white dot. The clumped, asymmetric appearance of cauda equina fibers and contrast enhancement indicates the existence of inflammation

Eighteen hours after delivery, the patient reported postural headache and stiff neck, consistent with PDPH. Her symptoms were refractory to conservative management, such as intravenous hydration and bed rest, as well as oral loxoprofen sodium. An AEBP was performed approximately 44 h after delivery using 20 mL of autologous blood injected at the L3–4 interspace with a median approach using a 17-gauge Tuohy needle. Back pain or neurologic symptoms were not noticed at this time. Symptoms transiently disappeared, but recurred 2 days later. Five days after delivery, a repeat AEBP was performed with 20 mL of autologous blood at the L2–3 interspace, where the UDP had occurred, using a paramedian approach. These procedures were performed by the most experienced anesthesiologists available, using loss-of-resistance to saline technique without difficulty. Blood was obtained using 10% povidone iodine skin preparation for each procedure. During the second AEBP, the patient reported pain in the back, buttocks, and posterior aspect of the lower extremities, as well as bilateral S1 radicular pain. Following the second AEBP, PDPH quickly resolved, but severe and transient symptoms were observed. The patient was unable to extend her legs beyond 135° due to radiating pain. No dysuria was present. MRI demonstrated intrathecally extending subdural hematoma around an aggregated cauda equina from L3 to L4 and another similar lesion at the L5 vertebral level (Figs. 1 and 2). No signs of epidural hematoma or infection were identified. Subdural hematoma and adhesive arachnoiditis were diagnosed and oral analgesic therapy was continued. Eight days later, a repeat MRI examination demonstrated partial improvements. One month after delivery, her residual neurologic symptoms included occasional discomfort in the right posterior thigh. The patient decided to request further evaluation only if symptoms were to worsen and declined further radiologic studies at the time.

## Discussion

We present a case of subacute subdural hematoma and arachnoiditis following two AEBP courses. Following the first uneventful treatment one interspace below, the repeat procedure at the interspace of UDP resulted in neurologic sequelae. We suspect that the blood pervaded through the preexisting dural hole into the subdural space. We reached this diagnosis with early neurologic examination and MRI, excluding possibilities of other treatable complications. Although this case involved an obstetric patient, the complication can happen in any patient who undergoes AEBP.

PDPH with epidural needles is reported to last at least 4–6 days without successful treatment [14]. Treatment options include hydration, caffeine, and oral analgesics, all of which are regarded as ineffective, compared to AEBP. In case of post-epidural PDPH in the obstetric population, complete and permanent relief with a single course of AEBP occurs in approximately 30% of patients [14]. Among patients who have undergone one failed AEBP, a similar success rate is expected for repeated treatment [14, 15].

It is unclear whether one failed AEBP should prompt repeated AEBP. The pierced dura eventually heals with fibrin deposition [16]. Additionally, following blood injection, the epidural space may become inflamed [17], making repeated interventions more difficult and potentially prone to complications; there are three reports of severe outcomes following repeated AEBP in an obstetric context [18–20]. Moreover, PDPH can become complicated by a headache of a different origin (e.g., psychological, vascular, or musculoskeletal) [21]; in such cases, AEBP is futile. Risks and benefits of repeated AEBP must be discussed beforehand.

To our knowledge, no study exists regarding the optimal volume of blood used in a repeated AEBP procedure. Moreover, this is unclear for the first AEBP, although a prior study supported injection of 20 mL [22]. In addition, the amount of blood may not significantly impact efficacy [23]. Therefore, we suspect that no rationale exists to inject a volume of blood in excess of 20 mL.

In our case, although the decision to perform repeated AEBP seemed reasonable, blood injection at the interspace of UDP may have been inappropriate. While supporting literature is insufficient [18, 24, 25], performing AEBP at the level of the dural hole may cause subdural blood infusion. No optimal location for AEBP has been established. The preferred method has been an approach at or one interspace below the dural injury, based on an imaging study favoring cephalad blood distribution [4]. Additionally, we found no reports of complicated AEBPs with blood injected below the interspace of UDP in the obstetric population (Table 1). It may be wise to avoid the interspace of UDP unless absolutely necessary.

**Table 1:** Previously reported obstetric cases with adverse events following autologous epidural blood patch

Reference	Age (years)		UDP or attempted procedure	Level of injury	AEBP	Level of AEBP	Outcomes
Aldrete, USA, 1997 [26]	34	LA	Multiple attempts at epidural placement	N/S	Prophylactic 19 mL from the indwelling catheter, which the author presumes to have been subdurally placed	N/S	Severe low back pain radiating toward both lower extremities, burning sensation in both feet, photophobia, and phonophobia Lumbar pain and burning sensation in the lower extremities 18 months later
Oh, USA, 1998 [27]	30	CS	Difficult spinal anesthesia	L2–3	Therapeutic 20 mL 5 days later	N/S	Fever, worsening headache, photophobia, and nuchal rigidity No neurologic sequelae 14 days later
Kalina, USA, 2004 [10]	27	LA	UDP with an epidural needle	N/S	Therapeutic 27 mL 4 days later	N/S	Severe back pain and radicular symptoms Symptoms gradually improved over several months
Riley, USA, 2009 [18]	39	ECV	UDP with an epidural needle	L3–4	Therapeutic 58 mL 1 day later	L3–4	Persistent back and right leg pain 1 week later Subdural hematoma on MRI Complete resolution of symptoms 2 weeks later
	33	LA	Multiple attempts at epidural placement	L4–5	Therapeutic 35 mL 1 day later 60 mL 2 days later 70 mL 4 days later	L3–4 L4–5 L2–3	Burning pain in the posterior buttocks and left thigh without numbness or weakness Segregation of the nerve roots and thecal sac on MRI Residual symptoms despite physiotherapy 6 months later
Verduzco, USA, 2011 [24]	37	PTL	Spinal anesthesia	L3–4	Therapeutic 20 mL 12 h later	L3–4	Neck discomfort that progressed over 10 days to the waist and both buttocks with radiation to the lateral aspect of both thighs No neurologic sequelae 5 weeks later
Devroe, Belgium, 2015 [17]	27	LA	UDP with an epidural needle Intrathecal catheter placed	N/S	Therapeutic 20 mL 4 days later	N/S	Back pain, lumbar muscle spasms radiating to both buttocks and legs, and fever No neurologic sequelae 14 days later
Carlswald, Sweden, 2015 [19]	29	LA	UDP with an epidural needle Intrathecal catheter placed	L3–4	Therapeutic 25 mL 36 h later 30 mL 2 days later	L2–3 N/S	Lumbar pain and radiculopathy in both legs as well as pain radiating to the upper thoracic region Wheelchair-bound 1 year later Arachnoiditis still demonstrable with MRI 2 years later
Hudman, UK, 2015 [20]	27	LA	UDP unrecognized Procedure aborted	N/S	Therapeutic One dose 2 days later Another dose 3 days later	N/S N/S	Lower back pain that radiated to the left leg, worsening over the next 2 days No neurologic sequelae 10 days later
Roy-Gash, France, 2017 [25]	24	LA	UDP with an epidural needle Intrathecal catheter placed	L3–4	Therapeutic 30 mL 3 days later	L3–4	Fever, shivering, stiff neck, intense lower back pain, bilateral leg pain, frontal postural headache, dizziness, and diaphoresis 7 days later No neurologic sequelae 14 days later

*AEBP* autologous epidural blood patch, *CS* cesarean section, *ECV* external cephalic version, *LA* labor analgesia, *MRI* magnetic resonance imaging, *N/S* not specified, *PTL* postpartum tubal ligation, *UDP* unintentional dural puncture

The scarcity of literature causes difficulty in quantifying severe neurologic outcomes of AEBP. To our knowledge, there are seven case reports of arachnoiditis after a single AEBP in an obstetric setting, with various neurological outcomes [10, 17, 18, 24–27] (Table 1). Severe outcomes are more likely when a large amount of blood pervades the dura, as indicated by either a larger amount of blood injected [10, 18, 19] or a subdural catheter used as a conduit [26].

In our case, MRI was useful because the clinical diagnosis was challenging. Subdural hematoma and adhesive arachnoiditis, which may be irreversible [19, 26], may mimic treatable complications, including abscess formation [13]. Early diagnosis is important to manage treatable complications promptly and to ensure follow-up. Fortunately, the patient’s condition improved within 1 month after the incident. Further studies are needed to elucidate the nature of these rare complications.

Although uncommon, subacute subdural hematoma and adhesive arachnoiditis can occur after AEBP, with potentially greater likelihood following a repeated procedure and if the procedure is performed on the interspace of UDP. Our case highlights that it is prudent to reconsider and discuss risks and benefits before performing repeated AEBP; it may be important to avoid the interspace of UDP, which may be correlated with severe adverse effects. A thorough neurological evaluation and MRI examination are warranted if a patient experiences persistent low back pain and radiculopathy following AEBP, which suggests subdural hematoma and adhesive arachnoiditis.

## Abbreviations

AEBP: Autologous epidural blood patch
CSEA: Combined spinal-epidural anesthesia
MRI: Magnetic resonance imaging
PDPH: Post-dural-puncture headache
UDP: Unintentional dural puncture
